# Reduced Graphene Oxide (rGO)-Loaded Metal-Oxide Nanofiber Gas Sensors: An Overview

**DOI:** 10.3390/s21041352

**Published:** 2021-02-14

**Authors:** Sanjit Manohar Majhi, Ali Mirzaei, Hyoun Woo Kim, Sang Sub Kim

**Affiliations:** 1 Division of Materials Science and Engineering, Hanyang University, Seoul 04763, Korea; sanjit.majhi@kaust.edu.sa (S.M.M.); hyounwoo@hanyang.ac.kr (H.W.K.); 2 The Research Institute of Industrial Science, Hanyang University, Seoul 04763, Korea; 3 Department of Materials Science and Engineering, Shiraz University of Technology, Shiraz 71557-13876, Iran; Mirzaei@sutech.ac.ir; 4 Department of Materials Science and Engineering, Inha University, Incheon 22212, Korea

**Keywords:** gas sensor, reduced graphene oxide, rGO-loading, metal oxide, sensing mechanism

## Abstract

Reduced graphene oxide (rGO) is a reduced form of graphene oxide used extensively in gas sensing applications. On the other hand, in its pristine form, graphene has shortages and is generally utilized in combination with other metal oxides to improve gas sensing capabilities. There are different ways of adding rGO to different metal oxides with various morphologies. This study focuses on rGO-loaded metal oxide nanofiber (NF) synthesized using an electrospinning method. Different amounts of rGO were added to the metal oxide precursors, and after electrospinning, the gas response is enhanced through different sensing mechanisms. This review paper discusses rGO-loaded metal oxide NFs gas sensors.

## 1. Introduction

**Figure 1:**
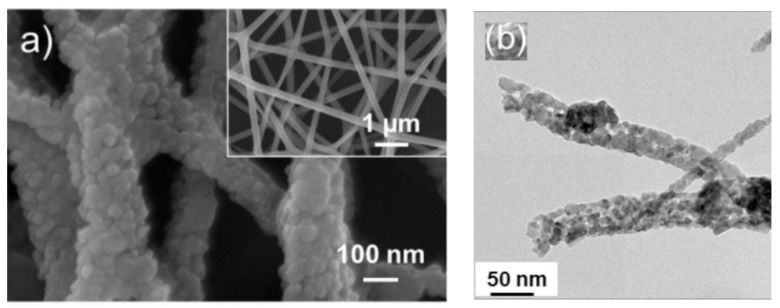
(**a**) FE-SEM image of ZnO electrospun NFs. The inset indicates high magnification image [20] (**b**) TEM image of 0.5 SnO_2_–0.5 Co_3_O_4_composite NFs [21].

Metal oxide gas sensors are used to sense various toxic gases and vapors [1,2] in many areas [3]. These sensors are quite popular owing to their low preparation costs, high sensitivity, fast dynamics, high stability, simple operation, and small size [4]. The general gas sensing mechanism of metal oxide gas sensors stems from the modulation of electrical resistance in the presence of target gases [5]. In these sensors, the sensing layer is exposed directly to the gas. The interaction between the target gas and sensing layer modulates the electrical resistance of the gas sensor, resulting in the generation of a sensing signal. This conductivity change is due to variations in the width of the electron depletion layer across the exposed area of the sensing layer [6]. Various strategies can be used to enhance the gas sensing characteristics of metal oxide-based gas sensors, such as UV light activation [7], fabrication of p-n, p-p and n-n heterojunctions [8], noble metal decoration [9],surface engineering, and the generation of structural defects [10]. Thus far, different morphologies of metal oxides, such as nanowires [11], nanotubes [12], nanorods [13], nanobelts [14], nanofibers (NFs), nanosheets [15], and hierarchical structures [16], have been used in gas sensing studies. This is because gas adsorption on the surface of gas sensing relates directly to the surface area of gas sensors and higher adsorption of gas means higher sensing signal. Thus, several studies have attempted to synthesize morphologies with a high surface area to increase the sensing performance of gas sensors. In addition, a porous morphology [17] and hollow structures [18,19] can be other useful techniques to increase the gas sensing properties. Therefore, metal oxide NFs are highly popular for sensing studies because of a very high surface area results from the one-dimensional morphology and the presence of nanograins on their surfaces, as shown in Figure 1. The generation of depletion layers on nanograins causes the development of potential barriers, which will be modulated during exposure to the target gases, contributing to the sensing signal generation.

**Figure 2:**
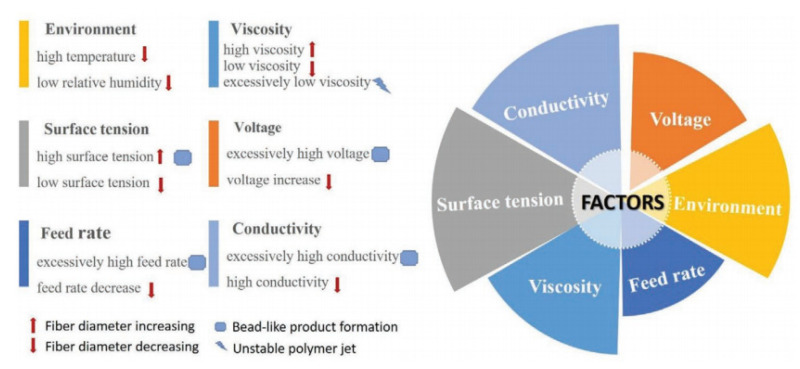
Factors affecting fiber formation during electrospinning [30].

Another advantage of NFs is their ease of preparation using a facile electrospinning technique. In general, the electrospinning technique can be used to fabricate continuous fibers with the possibility of control over the fiber diameter [22]. In addition, different NFs, such as porous NFs [23], core-shell NFs [24,25], and complex NFs [26], can be easily synthesized by electrospinning. Furthermore, NFs with controllable alignment can be produced by modifying the electrospinning [27]. Electrospinning also can be used for the mass production of NFs because of the easy handling, possibility of control of the diameter, low cost, simple operation, and high reproducibility [28]. Briefly, the features of electrospun fibers can be controlled by electrospinning parameters, including the solution variables (e.g., surface tension, viscosity, and conductivity), operating variables (e.g., applied voltage, spinning distance, and solution flow rate), and ambient variables (e.g., humidity and temperature) [29].Li et al. [30] (Figure 2) and Xue et al. [31] reviewed these factors, so this review paper does not discuss them further.

**Figure 3:**
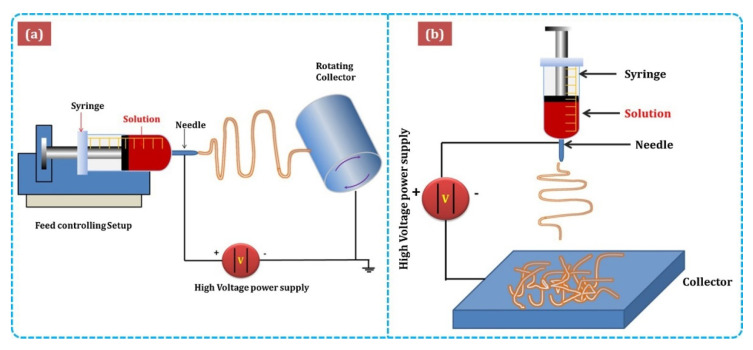
Schematic diagram of (**a**)horizontal and(**b**)vertical setups of electrospinning [34].

Figure 3 shows the major components of electrospinning include a syringe pump, spinneret, conductive collector, and power supply. The electrospinning technique can be described as follows: (1) charging of the liquid droplet and the formation of a Taylor cone, (2) formation of the charged jet, (3) thinning of the jet by the applied voltage, and (4) collection and solidification of the jet on a grounded collector. A Taylor cone is formed owing to surface tension and the applied electric field, from which a charged jet is ejected. The ejected jet was solidified quickly, resulting in the collection of solid fibers on the collector [32,33].

## 2. Graphene, Graphene Oxide, and Reduced Graphene Oxide

**Figure 4:**
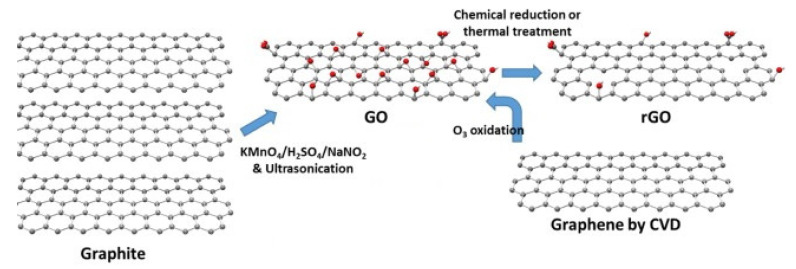
Structure and preparation of GO and rGO [44].

Graphene is a single-layer comprised of sp^2^carbon atoms that can be used as a gas sensor owing to its high charge carrier mobility (200,000 cm^2^ V^−1^ s^−1^), high mechanical stiffness, high environmental compatibility, and huge surface area (2630 m^2^g^−1^) [35,36]. Schedin et al. [37] introduced the first graphene gas sensor in 2007. Because atoms in a single layer of graphene can be considered surface atoms, graphene can interact with even a single molecule [37]. Even though nowadays pristine graphene can be synthesized on a large scale [38] with good water solubility [39,40], it can be easily agglomerated in solution due to surface interactions [41]. Moreover, graphene has no bandgap or functional groups, limiting its gas sensors applications, particularly in the pristine form [42]. Therefore, reduced graphene oxide (rGO), which is synthesized by the reduction of graphene oxide (GO), is a better choice for gas sensing applications because it has many functional groups and defects [43]. GO has also been used for gas sensing studies [44]. On the other hand, GO has very high resistance due to the presence of alkoxy (C-O-C), hydroxyl (-OH), carboxylic acid (-COOH), carbonyl (C=O), and other oxygen-based functional groups [45,46]. rGO has more defects and dangling bonds than graphite, resulting in better sensing properties [47]. GO is widely prepared using either Hummers [48] or Brodie [49] methods. In these methods, differences are in both the acid used (nitric or sulfuric acid), and the type of salt used (potassium chlorate or potassium permanganate). By subsequent reduction of GO, rGO can be obtained. In fact, rGO can be prepared easily from GO by chemical reduction, thermal reduction, and UV light reduction [50]. Figure 4 shows the structure and preparation of GO and rGO [44].

Furthermore, rGO has high thermal stability, and the total weight loss of rGO was reported to be only 11% up to 800 °C, which was attributed to the absence of most oxygen functional groups. [51]. Pristine rGO gas sensors have a long response and recovery times, and incorporating rGO with metal oxides can be a good strategy to increase the sensing capabilities of rGO-based gas sensors [52]. The synthesis and properties of rGO have been reviewed comprehensively [41]. The following section explains the gas sensing capability of rGO-loaded metal oxide NFs. This paper does not discuss the combination of rGO with other materials, such as mesh fabric [53] or polymers [54,55], for gas sensing studies. Furthermore, composites of metal oxides-rGO in morphologies other than NFs are not discussed.

### 2.1. RGO-Loaded Metal Oxides Gas Sensors

#### 2.1.1. rGO-Loaded ZnO NFs

Zinc oxide (ZnO) is one of the most common metal oxides in the gas sensing area because of its unique properties, such as n-type conductivity, low toxicity, ease of synthesis, high availability, good thermal stability, and high mobility of electrons [56,57]. Abideen et al. [58] prepared rGO-loaded ZnO NFs for H_2_ gas sensing investigations. At 400 °C, the sensor exhibited an exceptionally strong response (R_a_/R_g_) of 2542 to 10 ppm H_2_ gas. The presence of rGO and a semiconductor (ZnO)-to metal (Zn) transition in a H_2_ atmosphere were the main reasons behind the enhanced response to H_2_ gas. They suggested that ZnO could be converted to metallic Zn in the presence of H_2_ gas because of the high-sensing temperature. Owing to the differences in the work functions of rGO, ZnO, and Zn, at equilibrium, potential barriers were generated at both rGO/Zn and Zn/ZnO interfaces. ZnO was more n-type because of the flow of electrons from the Zn layer to ZnO. Accordingly, high resistance modulation occurred, contributing to the sensing signal. In addition, the potential barriers of rGO/Zn prevented the flow of electrons to rGO, which acted as a source of resistance modulation in the presence of H_2_ gas.

**Figure 5:**
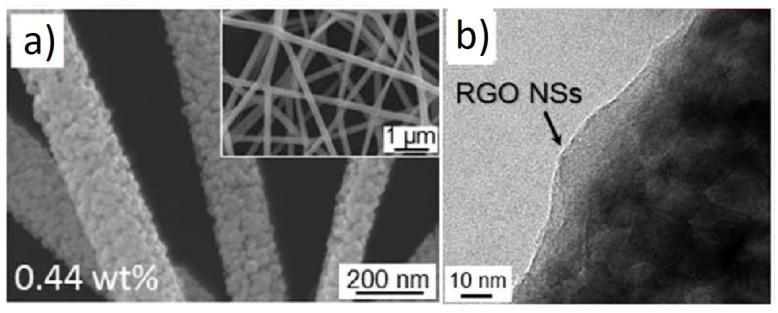
(**a**) SEM image and (**b**) TEM image of 0.44 wt.% rGO-loaded ZnO NFs [59].

In another similar investigation, Abideen et al. reported the effects of the rGO-loading (0.04, 0.11, 0.17, 0.44, 0.77, and 1.04 wt.%) on the NO_2_ sensing response of ZnO NFs [59]. Figure 5a,b shows SEM and TEM images of 0.44 wt.% rGO-loaded ZnO NFs, respectively.

**Figure 6:**
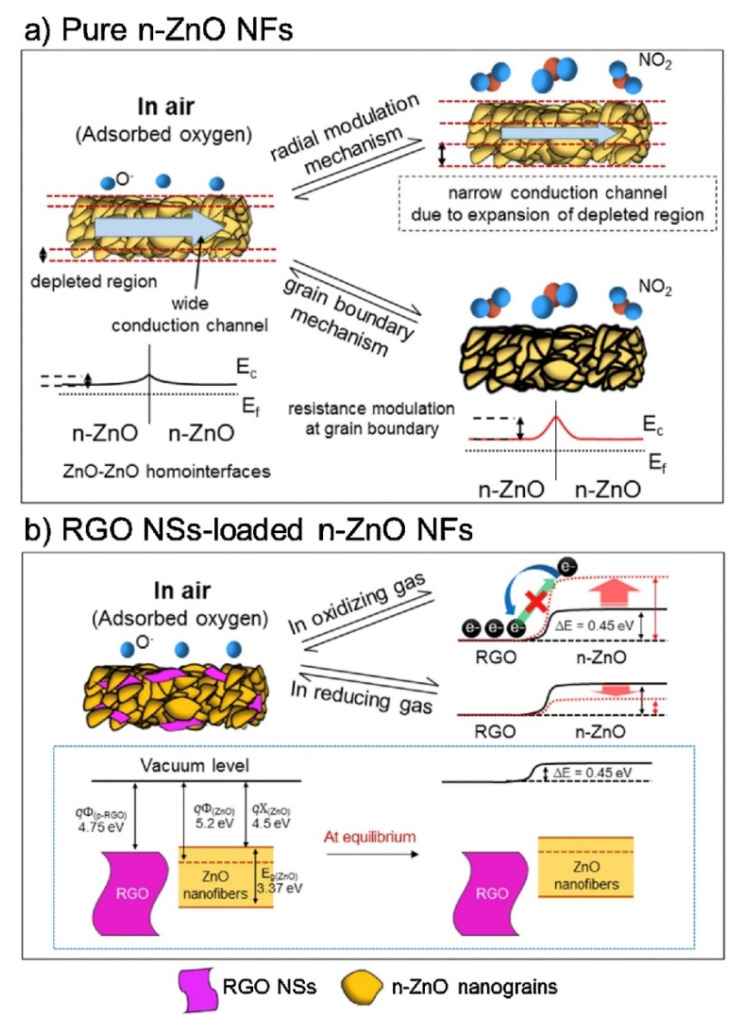
NO_2_ gas sensing mechanism of (**a**) ZnO NFs and (**b**) rGO-loaded ZnO NFs [59].

Among rGO-loaded ZnO NF gas sensors, the sensor with a 0.44 wt.% rGO-loading showed an enhanced response to NO_2_ gas. The sensor showed a response (R_a_/R_g_) of ~123 to 5 ppm NO_2_ gas at 400 °C.The boundaries between the nanograins acted as a source of resistance modulation caused by the generation of potential barriers resulting from oxygen adsorption. After introducing NO_2_ gas, the height of the potential barriers increased across the grain boundaries, leading to the sensor signal (Figure 6a).

In the rGO/ZnO heterojunctions, Ohmic contact was generated (Figure 6b), acting as a non-rectifying barrier to charge transfer, which affected the sensing behavior. In a NO_2_ atmosphere, the resistance of the gas sensor was increased because of the abstraction of more electrons. In addition, discretely distributed rGO nanosheets, which had a high surface area, played a catalytic role on NO_2_gas molecules. Other contributions were the presence of defects in rGO, which were favorable sites for the adsorption of gases.

**Figure 7:**
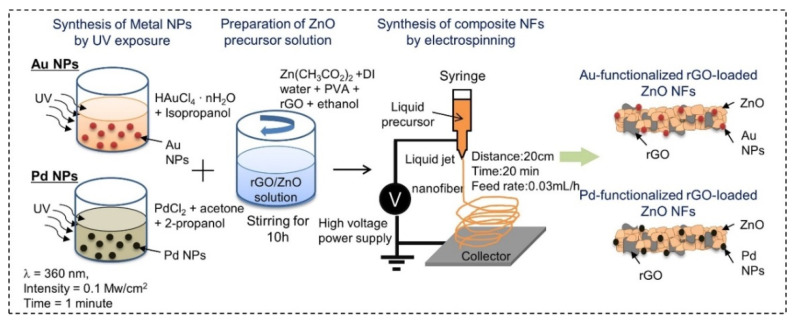
Preparation of Au and Pd-decorated rGO-loaded ZnO NFs [60].

**Figure 8:**
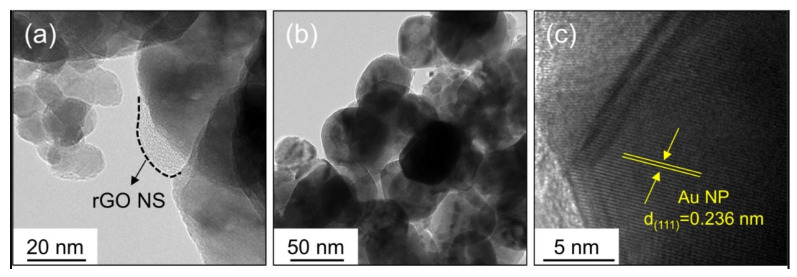
(**a**)TEM imageof Au-functionalized rGO-loaded ZnO NF. (**b**) TEM image of a ZnO NF. (**c**) Lattice-resolved TEM image of Au-functionalized rGO-loaded ZnO NF [60].

Abideen et al. synthesized rGO-loaded ZnO NFs functionalized by Au and Pd NPs, as shown in Figure 7 [60]. Figure 8a–c shows typical TEM images of rGO nanosheets, ZnO NFs and Au-decorated ZnO NFs, respectively. At 400 °C, the sensors functionalized with Au and Pd showed an enhanced gas response towards CO and C_6_H_6_ gases, respectively. Owing to the formation of ZnO/rGO heterojunctions, the electron depletion layer was wider in the ZnO NFs. Upon exposure to reducing gases, they interacted with oxygen species and liberated electrons, resulting in increased sensor conductivity, contributing to the sensing signal.

**Figure 9:**
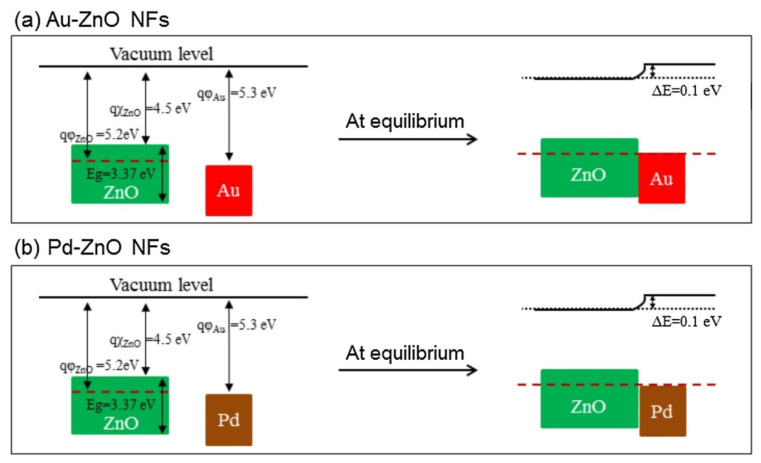
Energy band levelsof (**a**) Au-ZnO and (**b**) Pd-ZnO [60].

rGO has a high surface area, many defects and functional groups, and a different work function than ZnO. These factors contributed to the sensing enhancement. The effects of noble metals were considered. In the case of electronic sensitization, oxidized Pd (PdO), which initially took electrons from ZnO, was reduced in the presence of C_6_H_6_. Accordingly, the space charge layer was relaxed by the return of electrons to the ZnO. In chemical sensitization, Au and Pd NPs acted as catalytic metals. The incoming oxygen species were adsorbed on the surface of Au or Pd, leading to the dissociation of oxygen and subsequent spillover to neighboring ZnO. This increased the initial width of the electron depletion layers, which acted as a source of resistance modulation in the presence of the target gases. Furthermore, Schottky barriers were formed in the interfaces between Au/ZnO and Pd/ZnO (Figure 9). The width of the electron depletion layer decreased because of the flow of electrons from ZnO to metal. Moreover, the width of the electron depletion layer decreased by subsequent interaction of reducing gases and the liberation of electrons, causing a large change in resistance of the gas sensor.

The good selectivity of the Au-functionalized gas sensor to CO was attributed to the low (1.20 eV) oxidation barrier of CO on Au and the strong interaction of Au with CO gas. In addition, strong adsorption of C_6_H_6_ on Pd resulted in the strong response of the Pd-functionalized gas sensor to benzene.

#### 2.1.2. rGO-Loaded SnO_2_ NFs

**Figure 10:**
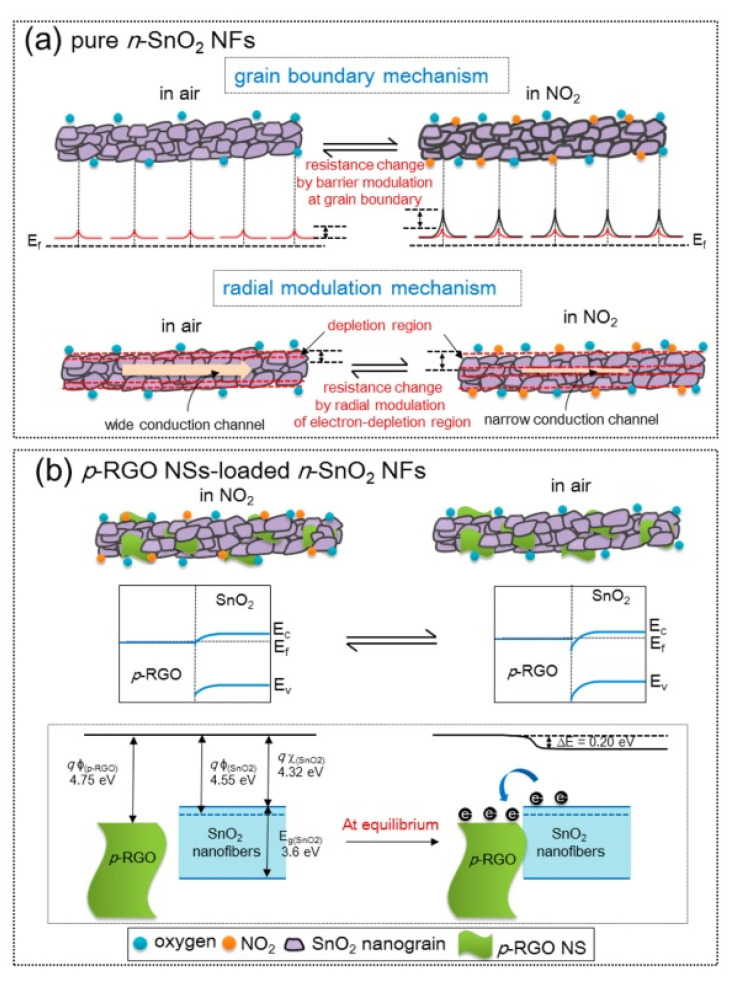
NO_2_ sensing mechanism in (**a**) SnO_2_ NFs and (**b**) rGO-loaded SnO_2_ NFs [63].

SnO_2_ is among the most popular metal oxides for sensing studies because of its high stability, favorable bandgap, low price, and good intrinsic sensing properties [61,62]. Lee et al. [63] examined the effects of rGO-loadings (0.04–1.04 wt.%) on the NO_2_ gas sensing properties of SnO_2_ NFs produced by electrospinning. The sensor with 0.44 wt.% rGO exhibited the highest resistance and the strongest response (R_a_/R_g_) of ~100 to 5 ppm NO_2_ gas at 200 °C. The pristine sensors showed a change in resistance due to the grain boundary mechanism and radial modulation, as shown in Figure 10a. For rGO-loaded sensors (Figure 10b), p-n heterojunctions were formed, generating potential barriers to the flow of electrons. The heights of these barriers were changed in the target gas atmosphere, resulting in sensing signal generation.

**Figure 11:**
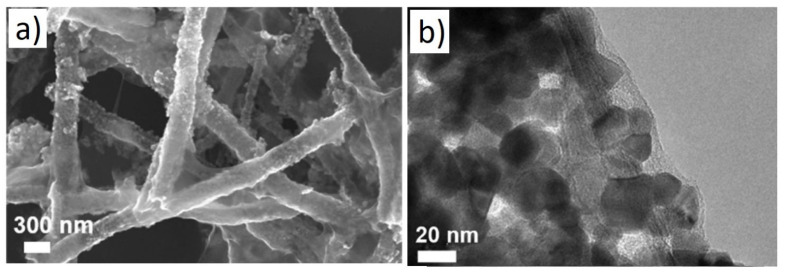
(**a**) SEM image and (**b**) TEM image of rGO-loaded SnO_2_ hollow NFs [65].

**Figure 12:**
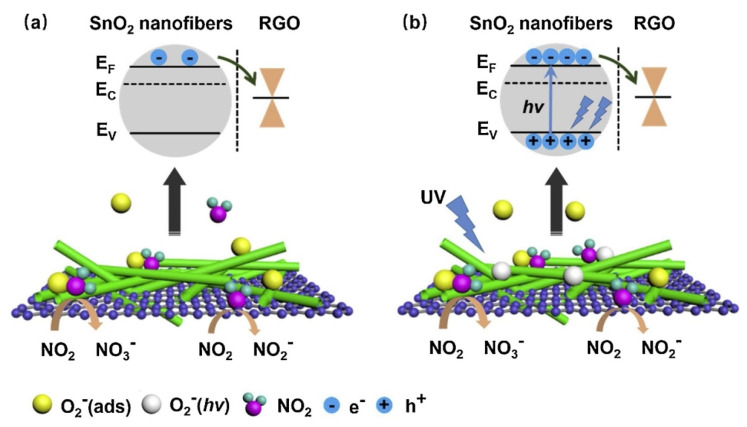
The sensing mechanism of rGO-loaded SnO_2_NFs to NO_2_(**a**) in the dark and (**b**) UV illumination [65].

Hollow NFs are also interesting morphologies for gas sensing studies because of their higher surface areas that can interact with gas species [64]. For example, a BET surface area of hollow SnO_2_NFs composited with GO was reported to be 33.4m^2^/g. In this regard, Li et al. [65] investigated rGO-loaded SnO_2_ NFs for gas sensing studies. Figure 11 shows typical SEM and TEM images of rGO-loaded SnO_2_ NFs.

p-n heterojunctions were formed between the connections owing to the intimate contact between rGO and SnO_2_. (Figure 12a). Under UV illumination (Figure 12b), SnO_2_acted as a UV absorber and a collector of generated electrons, whereas rGO acted as a photoelectron acceptor and provided many electron transport pathways. Generated electrons, as a result of UV illumination, can be adsorbed by oxygen species. The holes generated can be combined with chemisorbed oxygen, resulting in the desorption of oxygen. Thus, equilibrium is achieved under UV light. The best response could be achieved by tuning the UV light intensity. Oxidizing gases, such as NO_2_, can abstract electrons from the surface of the gas sensor. Compared to the dark condition, many more photoelectrons are captured by NO_2_ gas, resulting in a stronger response to NO_2_ gas. The strongest response was obtained under a 97 mW/cm^2^ UV light intensity, which indicated a response of ~100% (ΔR/R_a_ ×100) to 5 ppm NO_2_ at room temperature.

Choi et al. [66] prepared 0.01 and 5 wt.% rGO-loaded SnO_2_ NFs for sensing studies. In the 0.01 wt.% rGO-loaded sensor, the electrical transport and sensing characteristics of the sensor were dominated by the SnO_2_ NFs, which showed an enhanced gas response to H_2_S. In contrast, in the sensor with 5 wt.% rGO, the rGO NSs showed an enhanced response to acetone. The 0.01 wt.% rGO NS-loaded SnO_2_ NFs showed a response (R_a_/R_g_) of 33.7 to 5 ppm H_2_S at 200 °C. On the other hand, the 5 wt.% rGO-loaded sensor showed a response of 10.4 to 5 ppm acetone at 350 °C. Accordingly, the selectivity of the gas sensor towards H_2_S or acetone could be tuned by changing the amount of rGO.

**Figure 13:**
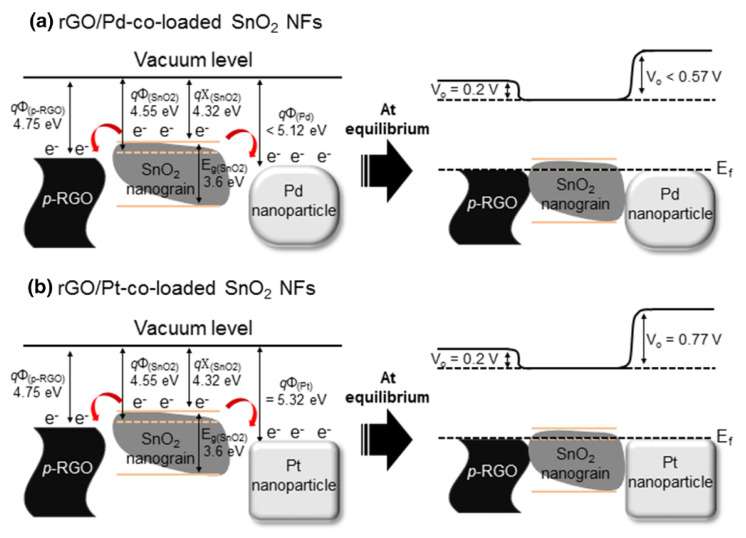
Energy band of (**a**) rGO, SnO_2_ Pd, and (**b**) rGO, SnO_2_ Pt [67].

Kim et al. [67] fabricated Pt or Pd rGO-co-loaded SnO_2_ NFs for sensing purposes. The sensors sensitized with Pt showed an enhanced gas response to toluene, while the sensor with Pd showed an improved response to benzene. Figure 13 shows the sensing mechanism. rGO plays a role in the formation of heterojunctions with SnO_2_, where electrons from SnO_2_ migrate to rGO, leading to an expansion of the electron depletion layer of SnO_2_ and a change in the width of the electron depletion layer in the presence of target gases, which greatly affect the response to gases. The sensing enhancement of the Pt-functionalized sensor was attributed partially to the H_2_ dissociation of gases. Upon dissociation, C_7_H_8_ can generate more H_2_ molecules. Thus, Pt dissociated C_7_H_8_ more effectively than the remaining gases. Furthermore, the methyl group (-CH_3_) on toluene made it easier to adsorb on the Pt surface. Regarding the Pd-functionalized gas sensor, the adsorption energy of C_6_H_6_/Pd (1.35 eV) was lower than that of C_6_H_6_/Pt (1.49 eV); thus, the Pd-functionalized gas sensor showed an enhanced response to C_6_H_6_ compared to C_7_H_8_ gas.

#### 2.1.3. rGO-Loaded α-Fe_2_O_3_ NFs

n-Type α-Fe_2_O_3_ is one of the most popular oxides, with many excellent features, such as low cost and facile preparation [68]. In that study, 0.5, 1.0, and 3.0wt.% rGO-loaded α-Fe_2_O_3_ NFs with approximate diameters of 100 nm were prepared using an electrospinning technique. The response of the optimal gas sensor, namely 1wt.% rGO/α-Fe_2_O_3_NFs at 375 °C to 100ppm acetone was approximately 8.9, which was approximately 4.5 times higher than the pure α-Fe_2_O_3_gas sensor [69]. The resistance of the composite sensors was lower than that of the pristine sensor, and the sensors with larger amounts of rGO showed lower conductivity. Ohmic contacts were generated because of the formation of rGO-Fe_2_O_3_ heterojunctions, which contributed to the sensing signal. Furthermore, defects and functional groups in rGO provided strong adsorption sites for gas molecules. In addition, the spaces between the layers of rGO nanosheets acted as an effective gas diffusion channel, which also provided more adsorption sites for gas molecules. When the amount of rGO was 3 wt.%, the surface of Fe_2_O_3_ was covered further with rGO, resulting in a significant decrease in the number of gas adsorption sites of α-Fe_2_O_3_. Thus, acetone gas reacted mainly with rGO rather than α-Fe_2_O_3_NFs, leading to a low response.

**Figure 14:**
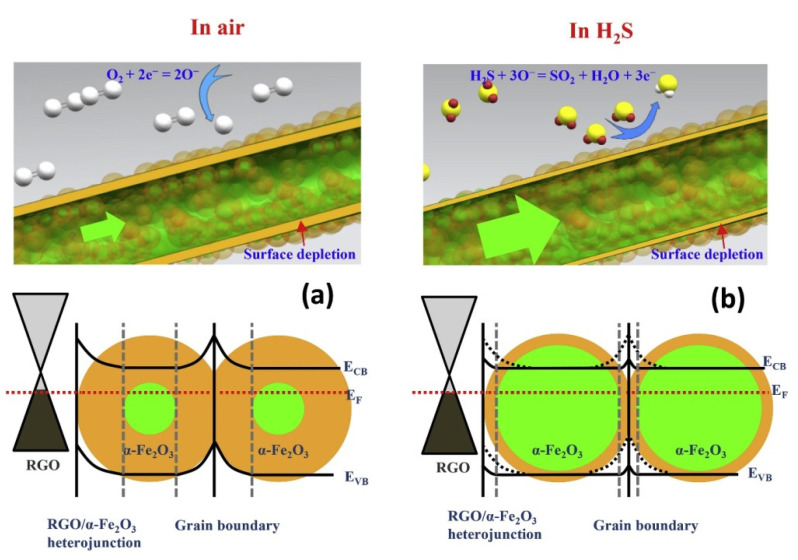
Schematic diagram of H_2_S sensing mechanisms of α-Fe_2_O_3_ NFs and rGO-loaded Fe_2_O_3_ NFs in (**a**) air and in (**b**) H_2_S gas [70].

Hoang et al. [70] prepared rGO-loaded (0–1.5wt.%) α-Fe_2_O_3_ NFs for sensing studies. The 1 wt.% rGO loaded sensor showed a strong response (R_a_/R_g_) of ~9.2 to 1ppm H_2_S gas at 350 °C. On the other hand, when rGO was increased, rGO nanosheets were dominant for the electron pathways, which decreased the overall sensor resistance, causing a weaker sensor response. The formation of rGO/Fe_2_O_3_ heterojunctions (Figure 14), high surface area resulting from the NFs, and strong adsorption sites, such as oxygen functional groups and structural defects, also enhanced the sensor response.

#### 2.1.4. rGO-Loaded In_2_O_3_ NFs

**Figure 15:**
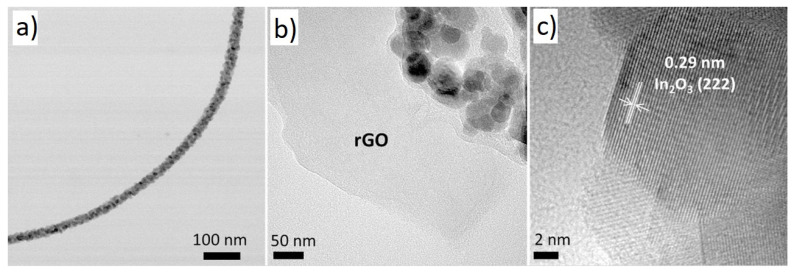
(**a**) Typical TEM image of rGO-loaded In_2_O_3_ NF. (**b**) TEM image showing rGO and (**c**) High-resolution TEM showing fringe of In_2_O_3_ [75].

As a semiconducting metal oxide, In_2_O_3_ (E_g_ = 3.6 eV) is used for sensing studies [71]. On the other hand, pristine In_2_O_3_ NFs show high resistance at room temperature because of the low mobility of electrons. A good strategy to overcome this problem is to form p-n heterojunctions and increase the overall sensing performance [72,73]. NH_3_is a highly reactive gas that harms the global greenhouse balance. Furthermore, it can be regarded as a biomarker of the human breath to diagnose lung diseases. Hence, the precise sensing of NH_3_gas is necessary [74]. Andre et al. synthesized rGO-loaded In_2_O_3_ NFs for gas sensing applications [75]. Figure 15 shows TEM analyses of rGO-loaded In_2_O_3_ NFs.

At room temperature, the sensor showed a response of 23.36 to 15 ppm NH_3_ gas. p-n heterojunctions of rGO-In_2_O_3_ were formed in the air upon intimate contact and exposure to NH_3_ gas; the electrons released greatly modulated the height of these potential barriers, resulting in the appearance of the sensing signal. Other factors that contributed to the sensing signal were the increase in surface area due to the presence of rGO nanosheets and the synergistic effect between the In_2_O_3_ structure and rGO nanosheets, which formed a three-dimensional interconnected morphology, facilitating the gas accessibility depth parts of the sensing layer.

**Figure 16:**
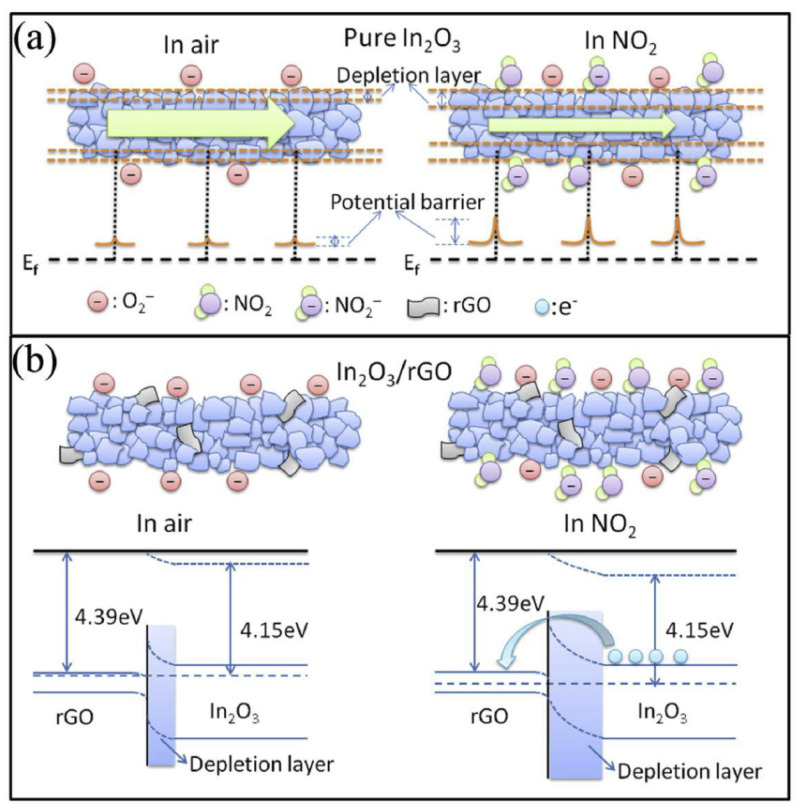
Gas sensing mechanism in (**a**) pristine In_2_O_3_ (**b**) rGO-loaded In_2_O_3_ [76].

Yan et al. [76] fabricated rGO-loaded In_2_O_3_ NFs by an electrospinning technique. The sensor with 2.2 wt.% rGO showed an enhanced gas response of 43 to 5 ppm NO_2_ gas at 50 °C. Figure 16a,b show the sensing mechanism for the pristine and rGO-loaded gas sensors, respectively. Several contributions were mentioned to explain the sensing mechanism in rGO-loaded In_2_O_3_ NFs. First, rGO with a high surface area and defects and functional groups provided many adsorption sites for incoming NO_2_ gas molecules. Furthermore, potential barriers were formed due to the formation of rGO-In_2_O_3_ heterojunctions, and the width and height of these barriers were modified upon exposure to NO_2_ gas, which changed the resistance of the gas sensor. On the other hand, the amount of rGO was not optimized in the fabricated sensor.

#### 2.1.5. rGO-Loaded Co_3_O_4_ NFs

Co_3_O_4_ is a p-type (E_g_ = 1.6–2.2 eV) semiconductor and is used for sensing different gases [77,78,79]. Feng et al. [80] synthesized the rGO-loaded Co_3_O_4_NFs using the electrospinning technique. At room temperature, the rGO-loaded sensor showed a 10-fold stronger response to NH_3_ than the pristine gas sensor. Ammonia is an electron donor gas that donates electrons to rGO upon adsorption. Therefore, the resistance of the gas sensor was increased. The rGO nanosheets interacted with the Co_3_O_4_to form Co-C bonds and therefore were polarized. Thus, the interaction with NH_3_, which had one lone pair of electrons, was stronger. The good selectivity was attributed to the different adsorption ability of different gases. Furthermore, NH_3_ is more polar than other tested gases, leading to better interactions with the sensing layer.

#### 2.1.6. rGO-Loaded CuO NFs

**Figure 17:**
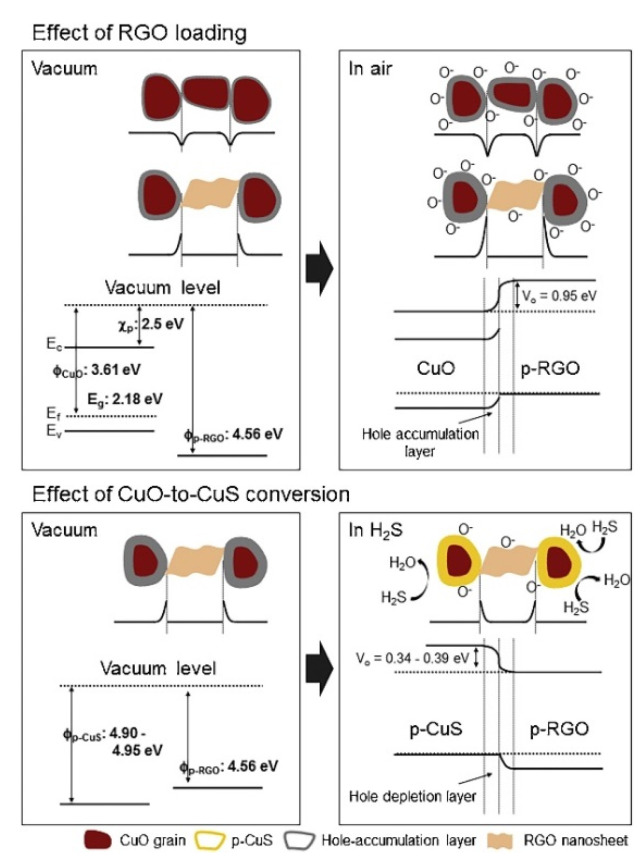
(upper part) Formation of rGO-CuO heterojunctions (lower part) conversion of CuO to CuS conversion [79].

CuO is a p-type metal oxide for H_2_S sensing, even for sub-ppb detection [81]. This is due mainly to the conversion of semiconducting CuO to CuS with metallic-like conductivity [82,83]. Kim et al. [84] used rGO (0.05–1.5 wt.%)-loaded CuO NFs for H_2_S gas sensing. The sensor with 0.5 wt.% rGO exhibited the maximum response of 1.95 to 10 ppm H_2_S at 300 °C. Figure 17 presents the sensing mechanism. Upon intimate contact between rGO and CuO, heterojunctions were formed, increasing the resistance of the gas sensor. In the H_2_S gas atmosphere, CuO was transformed into the CuS phase according to the following reaction:

CuO(s) + H_2_S(g) → CuS (s) + H_2_O(g) (1)

Accordingly, the CuS with metallic conductivity was formed, which led to a significant change in the potential barrier height and decreased the hole accumulation layer, contributing to the sensing signal. rGO nanosheets were separated by CuO crystals, and there was no direct contact among the rGO nanosheets. For small amounts of rGO, the current flowing through CuO was blocked by the presence of discrete rGOs, which acted as a source of modulation. On the other hand, for a high content (>0.5 wt.%) of rGO, the rGO nanosheets provided additional paths for current flow, leading to a higher conduction path along rGO, which reduced the initial resistance and the response of the sensor. The promising roles of rGO nanosheets were also attributed to defects and oxygen functional groups on rGO, which provided many adsorption sites for H_2_S gas molecules. Furthermore, with the high surface area of rGO and spillover effect of rGO, H_2_S molecules were dissociated on rGO and spilled over to CuO crystals, leading to an increase in the gas response.

#### 2.1.7. rGO-Loaded ZnFe_2_O_4_ NFs

**Figure 18:**
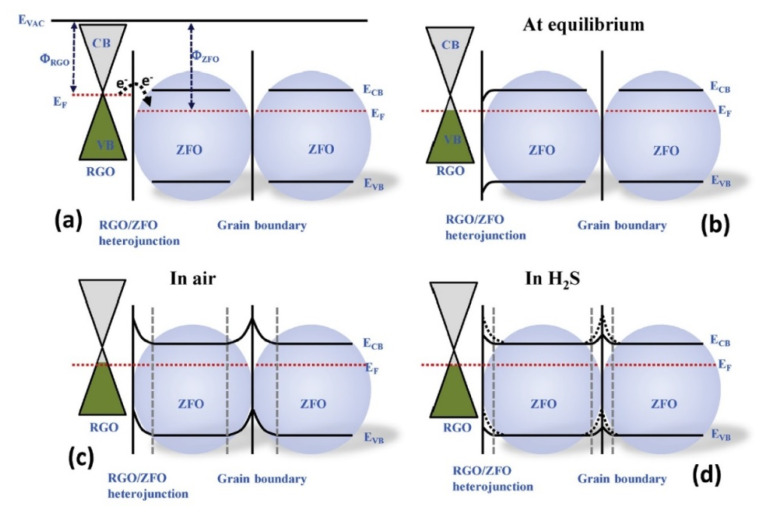
Band energy levels of (**a**) rGO and ZFO, (**b**) at equilibrium, (**c**) in air, and (**d**) in H_2_S gas [82].

Spinel ZnFe_2_O_4_ (ZFO) is an n-type metal oxide with a normal spinel structure [85] that has gained considerable attention for the detection of gases [86]. Hoang et al. [87] synthesized rGO-loaded ZFO NFs (50–100 nm) for H_2_S gas sensing applications. The sensor showed a response of 147 to 1 ppm of H_2_S gas at 350 °C. Figure 18 shows the mechanism of gas detection. Electrons from rGO were transferred to ZFO owing to different work functions, forming heterojunctions on the rGO-ZFO interfaces. Furthermore, potential barriers were formed along the grain boundaries of ZFO due to the adsorption of oxygen molecules. Upon the introduction of H_2_S gas, the electrons generated from the interaction of H_2_S with oxygen molecules, returned to the surface of the gas sensor, decreasing the barrier height, leading to the sensing signal.

Table 1 lists the gas sensing characteristics of rGO-loaded metal oxide NFs reported in literature. Different oxidizing (NO_2_) and reducing (C_6_H_6_, H_2_, CO, H_2_S, C_3_H_6_O, and C_7_H_8_) gases, have been successfully detected by these gas sensors. The sensing temperatures ranging from room temperature up to 400 °C have been reported. This demonstrates the possibility of a high sensing temperature of rGO-loaded gas sensors. In some cases, such as Ref. [58], a very high response to target gas can be obtained. Finally, in some cases, high responses to low concentrations of gases have been reported. Overall, the data in Table 1 shows the promising effects of rGO to be used along with metal oxide NFs for gas sensing studies.

Also, Table 2 summarizes precursors, NF diameter, surface area and porosity of different rGO-loaded NF gas sensors reported in the literature. In all cases, initially, a viscous solution of precursor materials was prepared and then it was loaded into a syringe and subsequently NFs were produced by electrospinning process. It should be noted that in most cases, rGO was reduced from the synthesized GO (via Hummers method). In most cases, the surface area is not mentioned, but it could reach to 78.57 m^2^/g. In addition, in most cases the NFs with different diameters and mesoporous nature can be obtained by electrospinning.

## 3. Conclusions and Outlook

This review discussed various aspects of the sensing mechanisms of rGO-loaded metal oxide NFs gas sensors. Different metal oxides, such as ZnO, SnO_2_, Co_3_O_4_, CuO, In_2_O_3_, and other metal oxides, were used in combination with rGO for gas sensing applications. Metal oxide NFs with a high surface area, resulting from long and continuous morphology of NFs, produced by electrospinning, are among the most popular choices for gas sensors. Furthermore, as shown in Figure 1, nanograins on the surface of NFs, can increase the surface area of NFs and act as a high resistance source for the gas sensors. Pristine rGO gas sensors generally show weaker sensing properties than metal oxides because of the long response time and recovery time, as well as the lower response. When loaded on metal oxides, rGO nanosheets can increase the overall sensing surface and adsorption sites, which leads to the generation of p-n or p-p heterojunctions with metal oxides and modulate the resistances of the gas sensors. Furthermore, defects in rGO, such as oxygen vacancies and functional groups, can provide strong adsorption sites for target gases. Generally, there is an optimal amount of rGO that can give the strongest gas response. When the amount of rGO is higher than the optimal value, the main current can pass from rGO, which leads to a weaker response. In addition, for larger amounts of rGO nanosheets, some agglomeration may occur, resulting in a weaker response of the gas sensor. There are some strategies, such as functionalization with noble metals, to increase the overall sensing performance of rGO-loaded metal oxide NFs. Therefore, rGO-noble metal co-loaded metal oxides can be quite effective for sensing studies. Further advances can help load rGO nanosheets on metal oxide composite NFs. For example, p-n heterojunctions between different metal oxides can be fabricated, and the rGO loading can increase the sensing performance further.

## Figures and Tables

**Table 1:** Gas sensing characteristics of rGO-loaded metal oxide NFs.

Sensing Material	Target Gas	Conc. (ppm)	Response (R_a_/R_g_)	T (°C)	Ref.
rGO-loaded ZnO NFs	H_2_	10	2542	400	[58]
rGO-loaded ZnO NFs	NO_2_	5	123	400	[59]
Au/rGO-loaded ZnO NFs	CO	5	35.8	400	[60]
Pd/rGO-loaded ZnO NFs	C_6_H_6_	5	22.8	400	[60]
0.44 wt.% rGO-loaded SnO_2_ NFs	NO_2_	5	100	200	[63]
rGO-loaded-SnO_2_ NFs under UV light	NO_2_	5	100%(ΔR/R_a_ × 100)	25	[65]
0.01 wt.% rGO-loaded-SnO_2_ NFs	H_2_S	5	34	200	[66]
5 wt.% rGO-loaded-SnO_2_ NFs	C_3_H_6_O	5	15	350	[66]
rGO/Pd co-loaded SnO_2_ NFs	C_6_H_6_	5	12.3	200	[67]
rGO/Pt co-loaded SnO_2_ NFs	C_7_H_8_	5	16	200	[67]
1 wt.% rGO-loaded Fe_2_O_3_ NFs	C_3_H_6_O	100	8.9	375	[69]
1 wt.% rGO-loaded Fe_2_O_3_ NFs	H_2_S	1	9.2	350	[70]
rGO-loaded In_2_O_3_ NFs	NH_3_	15	23.37	25	[75]
2.2 wt.% rGO-loaded In_2_O_3_ NFs	NO_2_	5	43	50	[76]
1wt.% rGO–Co_3_O_4_NFs	NH_3_	50	53.6%(ΔR/R_a_) × 100	25	[80]
0.5 wt.% rGO-loaded CuO NFs	H_2_S	10	1.95	300	[84]
rGO-loaded ZnFe_2_O_4_ NFs	H_2_S	1	147	350	[87]

**Table 2:** Precursors, NF diameter, surface area and porosity nature of rGO-loaded NF gas sensors reported in literature.

Sensing Material	Precursors	NF Diameter (nm)	Surface Area (m^2^/g)	Porosity Type	Ref.
rGO-loaded ZnO NFs	Zinc acetate, PVA	190	NA	NA	[58]
rGO-loaded ZnO NFs	Zinc acetate, polyvinyl alcohol (PVA)	~150	NA	NA	[59]
Au and Pd/rGO-loaded ZnO NFs	Zinc acetate, PVA, HAuCl_4_⋅nH_2_O, PdCl_2_	~200	NA	Mesoporous	[60]
0.44 wt.% rGO-loaded SnO_2_ NFs	SnCl_2_.2H_2_O, polyvinyl acetate (PVAc)	~180	7.0574	Mesoporous	[63]
rGO-loaded-SnO_2_ NFs under UV light	SnCl_2_.2H_2_O, PVP, dimethyl formamide (DMF)	80–250	NA	NA	[65]
0.01 wt.% rGO-loaded-SnO_2_ NFs	PVP, polymethylmethacrylate (PMMA) + tin(IV) acetate, acetic acid	370	NA	NA	[66]
rGO/Pt and Pd co-loaded SnO_2_ NFs	SnCl_2_.2H_2_O, PVAc, PdCl_2_, H_2_PtCl_6_.nH_2_O, DMF	NA	NA	Mesoporous	[67]
1 wt.% rGO-loaded Fe_2_O_3_ NFs	Ferric acetylacetonte PVP	100	NA	NA	[69]
1 wt.% rGO-loaded Fe_2_O_3_ NFs	PVA, Fe(NO_3_)_3_.9H_2_O	50–100	NA	NA	[70]
rGO-loaded In_2_O_3_ NFs	InCl_3_, PVP, Monomethylamine (MMA), Trimethylamine (TMA), Triethylamine (TEA), and N,N-Dimethylformamide (DMF)	50	NA	Mesoporous	[75]
2.2 wt.% rGO-loaded In_2_O_3_ NFs	PVP, DMF and (In(NO_3_)_3_·4.5H_2_O	76	39.82	Mesoporous	[76]
1wt.% rGO–Co_3_O_4_NFs	Co(NO_3_)_2_·6H_2_O, PVP, Ethanol	200–300	78.57	Mesoporous	[80]
0.5 wt.% rGO-loaded CuO NFs	PVA, (Cu (CH_3_CO_2_)_2_)	∼50	NA	NA	[84]
rGO-loaded ZnFe_2_O_4_ NFs	Zn(CH_3_COO)_2_·2H_2_O, Fe(NO_3_)_3_·9H_2_O and PVA	50–100	NA	Mesoporous	[87]

NA: not available.

## Data Availability

Not applicable.
